# The Better Care Plan: a blueprint for improving America's healthcare system

**DOI:** 10.1093/haschl/qxad007

**Published:** 2023-06-20

**Authors:** Stephen M Shortell, John S Toussaint, George C Halvorson, Jon M Kingsdale, Richard M Scheffler, Allyson Y Schwartz, Peter A Wadsworth, Gail Wilensky

**Affiliations:** UC-Berkeley, 2121 Berkeley Way West, Berkeley, CA 94704, United States; Catalysis, Inc. 3825 East Calumet Street, Suite 400-114, Appleton, WI 54915, United States; The Institute for Intergroup Understanding, 1300 Bracketts Point Road, Wayzata, MN 55391, United States; Brown University, Providence, RI, United States; UC-Berkeley, 2121 Berkeley Way West, Berkeley, CA 94704, United States; FTI Consulting, 555 12th Street, Washington, DC 20004, United States; Amory Associates, 1310 Norwest Drive, Norwood, MA 02062, United States; Project Hope, 1220 19th Street, Washington, DC 20036, United States

**Keywords:** Healthcare reform, prospective payment, continuous quality improvement, patient safery and outcomes reporting

## Abstract

The United States falls far short of its potential for delivering care that is effective, efficient, safe, timely, patient-centered, and equitable. We put forward the Better Care Plan, an overarching blueprint to address the flaws in our current system. The plan calls for continuously improving care, moving all payers to risk-adjusted prospective payment, and creating national entities for collecting, analyzing, and reporting patient safety and quality-of-care outcomes data. A number of recommendations are made to achieve these goals.

## Teaser

There have been many and ongoing efforts to improve the performance of the US healthcare system. As a group of healthcare leaders, policymakers, and academic researchers, we share our expertise, experience, and thinking in developing an overarching blueprint to improve our nation's healthcare system. We call this blueprint the Better Care Plan. It is anchored in seven overall design principles and seven criteria to be certified as a Better Care Team. We provide evidence in support of these principles and criteria and make a number of recommendations to get started on the challenges facing stakeholders in implementing the Better Care Plan nationwide.

## Introduction

While there is much to be proud of in America's healthcare system, the flaws of the system are significant and stubbornly resistant to change. Care is expensive, fragmented, highly variable in quality, and too often unsafe. We need to change how we provide care, pay for it, and how we measure and report on the care provided.

Some progress has been made in improving risk-adjusted mortality, complication rates, and morbidity since publication over 20 years ago of two seminal Institute of Medicine reports: To Err is Human^1^ and Crossing the Quality Chasm.^2,3^ Yet, recent research suggests that nearly 25% of hospital admissions result in an adverse safety event of which nearly a third are serious.^4^ Further, there remain up to three-fold differences in hospital mortality and death from serious treatable conditions.^5^ Disparities are particularly prevalent in minority, low-income, and rural communities. Arkansas, for example, has a maternal mortality rate of 45.9 per 100 000 live births versus 11.7 in California.^6^ Most recently, the coronavirus disease 2019 (COVID-19) pandemic has shown us that crossing the quality chasm is a much longer leap for some people than others.

Fee-for-service payment remains dominant. It is largely reactive based on one-off transactions between patients and providers. Fee-for-service pays for the episodes of care provided by individual clinicians and not for the collective coordinated package of care provided by teams that work together. We cannot improve our nation's healthcare system by continuing to pay for the volume of care produced under our deeply rooted fee-for-service system.

We also cannot improve our nation's health without developing credible, transparent, standardized, validated, timely, and understandable patient safety and quality outcomes reporting. We need to build on the process measures developed to date by focusing on measures of patient harm and other outcomes.^7^ Consumers need this information for choosing health plans and plans and providers need it to continuously improve care, patient outcomes, and patient safety.

This paper puts forward principles and criteria of a Better Care Plan (BCP) to address the flaws in our current system and to serve as a foundation for continuous improvement. The BCP provides a blueprint for ensuring that universal coverage is affordable and equitable, with continuously improving care that can be sustained over time.

## Better Care Plan design principles

The BCP is anchored in three actions: to reorganize care to be continuously improving and patient-centered, to expand risk-adjusted prospective payments, and to make publicly available patient safety and outcome quality-of-care data. The plan is intended for all payers and provider organizations. To accomplish this, the BCP is guided by seven design principles.

To change how we organize and deliver care, there should be

1. integrated and coordinated team-based, technology-enabled, patient-centered primary care;
2. continuous improvement in care;
3. continuous efforts to eliminate inequities in care.

To change how we pay for care, there should be

4 risk-adjusted prospective payment to provider organizations.

To change how we report on patient safety and outcomes of care, there should be

5 patient access to personal health records and information on plan/provider organization performance;
6 transparency and accountability of health system patient safety and quality-of-care outcome performance measures for use by consumers, purchasers, and those held accountable for continuously improving care.

To recognize competitive forces in the US healthcare markets

7 competition should be based on patient safety and quality, access, and price.

### Team-based care

Team-based care is central to providing better care. Provider organizations that invest more in team-based primary care have better performance on measures of clinical quality, patient experience, utilization, and cost.^8^ Patients have increased odds of complications and death when teamwork is not present.^9^ A recent meta-analysis of thirty-one teams found positive effects on performance across multiple tasks, including postoperative complications and bloodstream infections.^10^ We propose that, to be certified as a Better Care Team, a provider organization will be required to do the following:

- Collaborate with patients in setting goals and developing care plans. Provide patients with ready access to their electronic health record (EHR) data and to performance measures of their providers.
- Coordinate care. This includes providing or arranging for all needed care, including preventive, specialty, urgent, behavioral, social, and mental healthcare, and actively coordinating that care across sites and providers over time.
- Be responsible for patient safety, health outcomes, and cost of care.
- Continuously improve care, with systems and training in place to make continuous quality improvement central to the group's culture.
- Provide access to point-of-care performance data and disseminate to providers.
- Work to ensure that every team member's competencies are fully utilized and that all are practicing at top of their license.
- Maintain current knowledge of the patient population in order to identify high-risk patients requiring special care and allocate resources based on need, vulnerability, and patient preferences, focusing on delivering timely care and eliminating disparities and inequities in care.

Better Care Teams will assess their effectiveness by promoting shared goals and mutual respect, and ensuring that communication is accurate, timely, frequent, and problem-focused.^11^ Better Care Teams will also work to achieve racial and ethnic diversity of staff and patient populations, educating staff on cultural competency, ensuring language access for non-English speakers, recognizing low health literacy and social needs of patients, and offering appropriate support services. For example, by creating individualized care plans that target resources needed for patients with multiple comorbidities, the Medicare Advantage Special Needs Plans (SNPs) have achieved significant reductions in hospital admissions.^12^

### Continuous quality improvement

Continuous quality improvement (CQI) is necessary to operate within the BCP. The core elements of CQI are that knowledge is grounded in data, care is team based, and care processes are always being investigated and improved with Plan-Do-Study-Act cycles. As of 2017, 69.3% of US hospitals report being engaged in continuous improvement projects, but only 12% report engagement across the entire organization.^13^ Therein lies the problem. CQI is a mindset for continuous learning, not a group of projects. The cultural norm should be to provide error-free care by ensuring that everyone is working to improve quality and patient safety every day.

CQI requires a method such as Lean, Lean Six Sigma, or Robust Process Improvement. Widespread use of CQI has been shown to be associated with higher patient experience scores, lower adjusted inpatient expense per admission, lower 30-day unplanned readmission rates, and less use of low-value care without a negative impact on overall mortality.^13^ In addition, significant declines in mortality rates from heart attacks and increases in patient satisfaction have been associated with CQI implementation^14^; there have been rapid changes in patient flow to improve outcomes during the COVID-19 pandemic^15^ and a marked decline in sepsis death rates from 20% to 3%.^16^ Using CQI methods, thousands of lives and millions of dollars could be saved in sepsis care alone.

CQI needs to become the norm. Actionable data must be available at the frontline in real time, management must support daily problem solving, and leadership must make patient safety and improving quality their top priority.

### Moving to risk-adjusted prospective payment

The weight of evidence suggests that healthcare organizations that assume more risk for the cost of care have significantly higher clinical quality of care and lower total cost of care than those that assume less risk or are paid only by fee-for-service.^17–19^ These organizations are more likely to have salaried primary care physicians, advanced data-management capabilities, preferred relationships with efficient specialists, and formal team-based programs to coordinate care for high-risk patients.^18^ Shared Savings Accountable Care Organizations (ACOs) at risk for the cost and quality of care provided are associated with lower costs on the order of 1.5% while maintaining or improving on selected quality-of-care measures.^19^ The longest existing commercial ACO initiative—the Massachusetts Blue Cross/Blue Shield Alternative Quality Contract—has shown ongoing savings over 8 years while maintaining or improving the quality of care provided.^20^ A systematic review of forty-six studies and related studies found that the risk-based, prospectively paid Medicare Advantage (MA) plans are associated with more preventive visits, fewer hospital admissions and emergency department visits, shorter lengths of stay, and lower spending than traditional Medicare, although no difference in readmission rates, mortality or racial/ethnic disparities.^21–26^ Recent research also shows that Medicare Advantage beneficiaries report better patient experience scores than Traditional Medicare Beneficairies.^27^ There is also evidence that increased market penetration of MA plans is associated with lower post acute care use by Traditional Medicare pateints without an increase in hospital readmissions.^28^

We support the Centers for Medicare & Medicaid Services’ (CMS’) and all payers’ efforts to move more rapidly toward establishing risk-adjusted prospective payments as the norm. In addition to creating incentives for prevention, keeping people well, and continuously improving needed care, prospective payment provides a predictable revenue stream and cash flow for provider organizations. This proved to be particularly helpful to providers during the COVID-19 pandemic.

Adjusting for differences in likely need for care should be based on audited encounter data as CMS is now doing.^29,30^ Adjustments should also be made for differences in the social determinants of health (food insecurity, housing instability, income and related) and for differences in the health status of historically disadvantaged groups,^31^ drawing on measures such as those used in the Area Deprivation Index.^32^

The transition to universal or near universal risk-adjusted prospective payment will take time as provider organizations continue to develop their capabilities to take on full financial risk for care. Time will also be needed for healthcare provider organizations to develop effective partnerships with community-based organizations that address the social determinants of health.^33^ We support CMS in their alignment strategy to work with all payers to address these challenges through models such as bundled payments that move toward full risk-adjusted prospective payments.^34^ Tailored compensation arrangements involving fee-for-service may still be needed for some specialty providers. These expenditures should be built into the provider organizations’ overall risk-adjusted prospective payment health budget.

### Patient safety and outcomes data reporting

Changing how care is delivered and paid for is necessary but not sufficient to continuously improve affordable care. Outcome measures are needed for accountability and to learn from and share evidence and best practices.

We suggest that a national task force be created with quality measurement, clinical, and communication science expertise and with payer, provider, and consumer input to create the needed measures and standards. This will involve (1) defining what will be measured; (2) mandating what will be reported; (3) developing a system that reduces the burden on human effort; (4) testing and refining; and (5) providing meaningful outcome data for patients, the general public, health plans, and providers alike.

Measurement should focus on risk-adjusted outcomes reported by race/ethnicity. A balance needs to be struck between a relatively limited number of clinically significant measures useful to help patients and purchasers evaluate and select health plans and providers and condition-specific, clinically significant measures such as HbA1c levels relevant for patients with diabetes. Patient-reported outcome measures should be incorporated as digital reporting evolves and a methodology is developed to ensure comparability among organizations. We support CMS’ building-block approach to aligning quality measures across all of their payment programs and for targeting 2030, if not sooner, as a completion date for creating a system for patient-reported outcomes.^35^

Once established, reporting should be mandated with national laws and regulations. The CMS Star Rating System used for Medicare Advantage plans is an example of a mandated reporting system used to incentivize quality and publicly report quality and patient experience measures making improvements over time.

The system should require minimal human interaction. The software should be able to extract measures from existing data sources. EHR companies have already developed robust observational databases that can serve this purpose. Interoperability is facilitated by the Fast Healthcare Interoperability Resources standards that enable seamless and secure healthcare information exchange, although implementation challenges must be addressed.^36,37^ Blockchains and artificial intelligence technology can minimize the administrative burden on health plans and provider organizations by facilitating data-mining collecting, aggregating, analyzing, and reporting patient safety and quality performance data.^38^

Rigorous testing and ongoing refinements will be needed. Measures might be classified into three categories: those under development in some early-adopting pilot sites, those being tested for fine-tuning, and then those ready for widespread adoption by all providers.

Easy-to-read health outcome and safety measures should be clearly stated on all sites that offer consumers a healthcare choice, from insurance exchanges to employer intranet sites and all organized care providers. Numerical grades, such as FICO credit scores, are one example of an effective way to highlight the outcome differences that exist across health plans and their associated provider organizations.

To facilitate the reporting of outcome data, we suggest creating a national patient safety and quality reporting system and repository. Such an entity is where data on, for example, inpatient falls and bedsores, adverse events, and actual-versus-expected mortality for conditions such as pneumonia, heart failure, and stroke are reported in real time by hospitals/health systems and become public record. This should include data on “near misses”.^39^ When a safety event occurs, investigating the problem and correcting the process at the point of care is the best chance to solve underlying issues.

## Implementation challenges

While some of the BCP principles and criteria have been implemented in some markets and areas of the country, there are a number of challenges for their nationwide adoption. Health plans, provider organizations, and others have been well rewarded by the longstanding entrenched fee-for-service payment system, encouraging the provision of largely acute and specialist-oriented care. The system is reactive and transactional, waiting for patients to arrive for care, not proactive and relational. The system is highly resistant to change. A century of habits instilled in medical and health professional education, practice, financing, and payment needs to be overcome. Below we offer a number of recommendations as a starting point, recognizing that a combination of policy “carrots and sticks” will be needed for widespread implementation.

## Recommendations

### Better Care certification

The key to providing better care is for providers to organize into patient-centered, technology-enabled, primary care teams to serve as the first point of access for patients, coordinate patient care over time, and take responsibility for the cost and outcomes of care. To do so, we recommend that an entity be established to certify health plans and provider organizations that meet BCP requirements.

### Increase primary care capacity

We recommend the following actions to increase the country's primary care capacity:

- Medical schools and related health-science professional schools should assure course content for students on teamwork, CQI processes, and completion of improvement projects. Continuing Medical Education (CME) programs should do likewise.
- Congress should act to increase the number of primary care physicians in the workforce by expanding incentives for students to choose primary care.^40^
- We support Senate Bill S.834, The Residency Shortage Reduction Act of 2021, to increase the number of residency slots including for hospitals located in rural areas and health professional shortage areas. Priority should be given to increasing the number of primary care physicians. Similar steps should be taken to increase training sites for nurse practitioners, nurses, physician assistants, medical social workers, clinical psychologists, and pharmacists.
- A national healthcare professional licensing body, working with the states and relevant professional associations, should be established to define common nationwide competency standards for professional practice for nurse practitioners, physician assistants, pharmacists, and other healthcare professionals so they can fully utilize their training in providing team-based care.

### Make risk-adjusted prospective payment the norm for paying for care

To align financial incentives with BCP quality objectives, all payers—Medicare, Medicaid, Children’s Health Insurance Program (CHIP), and commercial health plans—should accelerate the movement to risk-adjusted prospective payment as the norm for paying for care replacing fee-for-service–based payment. Actions to be taken include the following:

- Hospitals/health systems should accept risk-adjusted prospective payments from all payers. Tertiary referral centers should accept bundled payments for highly specialized procedures and episodes of care.
- Medicare should continue to use risk-adjusted (using audited encounter data) prospective payment for the Medicare Advantage program with accountability and incentives for quality and move Traditional Medicare to risk-adjusted prospective payment.
- States should require Medicaid managed-care plans to use risk-adjusted prospective payments to providers and encourage the availability of Medicare SNPs for beneficiaries who are dually eligible for Medicare and Medicaid.
- Employers of fifty or more employees should offer at least one health plan to their employees based on the BCP design principles and criteria.
- Medicare and Medicaid beneficiaries should be able to compare all of their plan choices side by side on enrollment information sites.
- States should ensure that price data be organized and available to employers, patients, and related third parties in an understandable way.

### Establish national patient safety and health outcomes reporting

The following actions should be taken to create a national accountability and reporting system:

- Congress should direct the Department of Health and Human Services (HHS) to establish and invest in a data-reporting protocol for all EHR vendors to enable the reporting of health outcomes by all payers that uses real-time data from EHRs, related claims-based data, and patient-reported outcome data.
- CMS should establish a national committee of clinicians, measurement experts, health plan representatives, and patient representatives that should be convened by the National Quality Forum, the National Committee on Quality Assurance (NCQA), or similar entity to define a small number of high-priority clinical outcomes that can be captured electronically and uploaded to established reporting sites on a daily basis. The data should be provided, analyzed, and reported by race/ethnicity and reported to facilitate comparisons of health plans and providers.
- Recognizing that diagnostic errors contribute to poor quality of care and patient harm, the National Quality Forum, NCQA, or similar entity should convene appropriate experts to develop relevant, reliable, and valid measures of diagnostic errors and recommend how these can be identified and reported on by provider organizations and clinical sites of care—both overall and by race/ethnicity.
- Outcomes data should be provided, analyzed, and reported by race/ethnicity and widely disseminated to facilitate comparison of health plans and provider organizations.

Specific to patient safety, we endorse House Bill H.R. 9377 creating a National Patient Safety Board.^41^ The Board should oversee an entity within HHS that houses a national repository of the best patient safety practices. The best-known patient safety practices from the highest performing health systems should be available to everyone online. The national repository would also be responsible for receiving all reports of serious patient harm, medical errors, “near misses,” and complications. The Board would use artificial intelligence to monitor and anticipate adverse events and then create recommendations for preventing such events.

An accrediting body such as the Joint Commission on Accreditation of Healthcare Organizations should be charged with overseeing that patient safety practices are being implemented and improved, and that each organization is complying with the patient safety reporting requirements.

## Conclusion

Changing how care is delivered and paid for go hand in hand. Expanding access to nationally reported patient safety and outcome data provides the critical feedback loop to know how well we are doing. It is time to move beyond the “pockets of excellence” that now exist within our healthcare system to the broad adoption of the BCP principles and criteria by all payers and provider organizations. Doing so will enable all Americans to receive continuously improving, more equitable, and affordable care.

## Supplementary Material

qxad007_Supplementary_Data
