# Supplementary material for: The Better Care Plan: a blueprint for improving America's healthcare system
Source: Health Aff Sch. 2023 Jun 20;1(1):qxad007. doi: 10.1093/haschl/qxad007 (PMC10986211; doi:10.1093/haschl/qxad007)
Supplement: qxad007_Supplementary_Data [file qxad007_Supplementary_Data.zip › BetterCare Policy Group.docx]

.

.

- .

.

.

.

# The Better Care Policy Group

The Better Care Policy Group is comprised of senior healthcare executives, policymakers, and researchers, who began meeting in mid-2020 to discuss the urgent needs of U.S. healthcare reform often with invited guests in leadership positions within the healthcare system. They have been holding bi-weekly discussions ever since. This paper is based on those discussions.

[George C. Halvorson](https://www.intergroupinstitute.org/about/team/george-halvorson) – Chair, Institute for InterGroup Understanding; Former CEO, Kaiser-Permanente Health Plans, Health Partners of Minnesota, and four other health care delivery and financing organizations; former Chair, The International Federation of Health Plans and The Partners for Quality Care.

[Jon M. Kingsdale, PhD.](https://www.linkedin.com/in/jon-kingsdale-3359043a/) –Associate Adjunct Professor at Tufts School of Medicine, and Brown University; Chair and interim President of the Atrius Health Equity Foundation; organized and led the Massachusetts Health Connector, the model for health insurance exchanges under Affordable Care Act (ACA).

.

[Richard M. Scheffler](https://publichealth.berkeley.edu/people/richard-scheffler/), PhD. – Distinguished Professor Emeritus of Health Economics and Public Policy, UC-Berkeley, Founding Director of the Nicholas C. Petris Center on Health Care Markets and Consumer Welfare.

[Allyson Y Schwartz](https://bettermedicarealliance.org/staff/allyson-y-schwartz/), MSS – Former member of the U.S. House of Representatives (PA); Senior Advisor, FTI Consulting; former President and CEO of the Better Medicare Alliance; member of RAND Health Advisory Board; Executive Advisory Board of the University of Pennsylvania Leonard Davis Institute for Health Economics, member of Academy of Medicine Culture and Inclusiveness Action Collaborative.

Stephen M. Shortell PhD, MBA, MPH – Distinguished Professor of Health Policy and Management Emeritus and Dean Emeritus, UC-Berkeley, Founding Director of the Center for Organizational and Innovation Research (CHOIR) at UC-Berkeley, member of the Board of Catalysis Inc. board member (liaison) of the Integrated Healthcare Association (IHA); member of the National Academy of Medicine; past editor of Health Services Research; past president of the Association for Health Services Research (Academy Health).

[John Toussaint, MD](https://createvalue.org/who-we-are/board/john-toussaint-md/) – Founder and Executive Chairman, Catalysis Inc.; adjunct Professor, Ohio State University’s Fisher College of Business; founding Chair of the Wisconsin Collaborative for Healthcare Quality and the Wisconsin Health Information Organization.

[Peter A. Wadsworth](https://www.linkedin.com/in/peterawadsworth/) – Managing Partner, Amory Associates; former healthcare investment banker and health insurance executive specializing in managed care.

[Gail Wilensky, PhD.](https://gailwilensky.com/) –former Administrator of Medicare and Medicaid and chair of the Medicare Payment Advisory Commission; member of the National Academy of Medicine; trustee, of the United Mine Workers of America’s Combined Benefits Fund, along with numerous non-profit and corporate boards.

# References
